# Renal Angiomylipoma with Epithelial Cyst (AMLEC): A Rare Cystic Variant of Angiomyolipoma

**DOI:** 10.5334/jbsr.3114

**Published:** 2023-04-24

**Authors:** Felix Delbare, Benjamin Leenknegt, Marc Lemmerling

**Affiliations:** 1 UZ Ghent, BE; 2 AZ Sint-Lucas Ghent, BE

**Keywords:** angiomyolipoma, angiomyolipoma with epithelial cyst, kidney, ultrasound, MRI

## Abstract

**Teaching Point:** AMLEC is a rare subtype of an angiomyolipoma (AML) and not a primary cystic lesion.

## Case History

**Figure 1:**
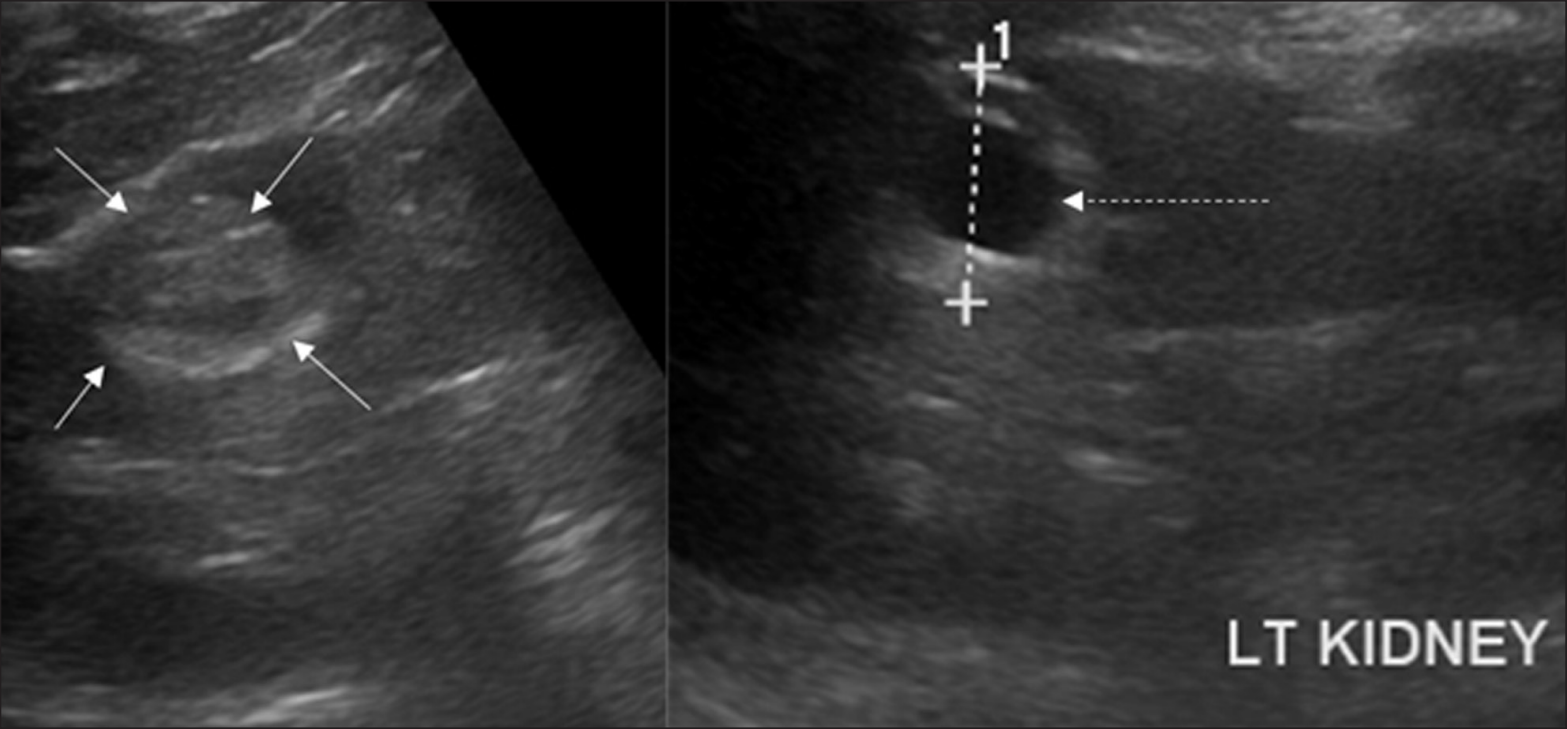


**Figure 2:**
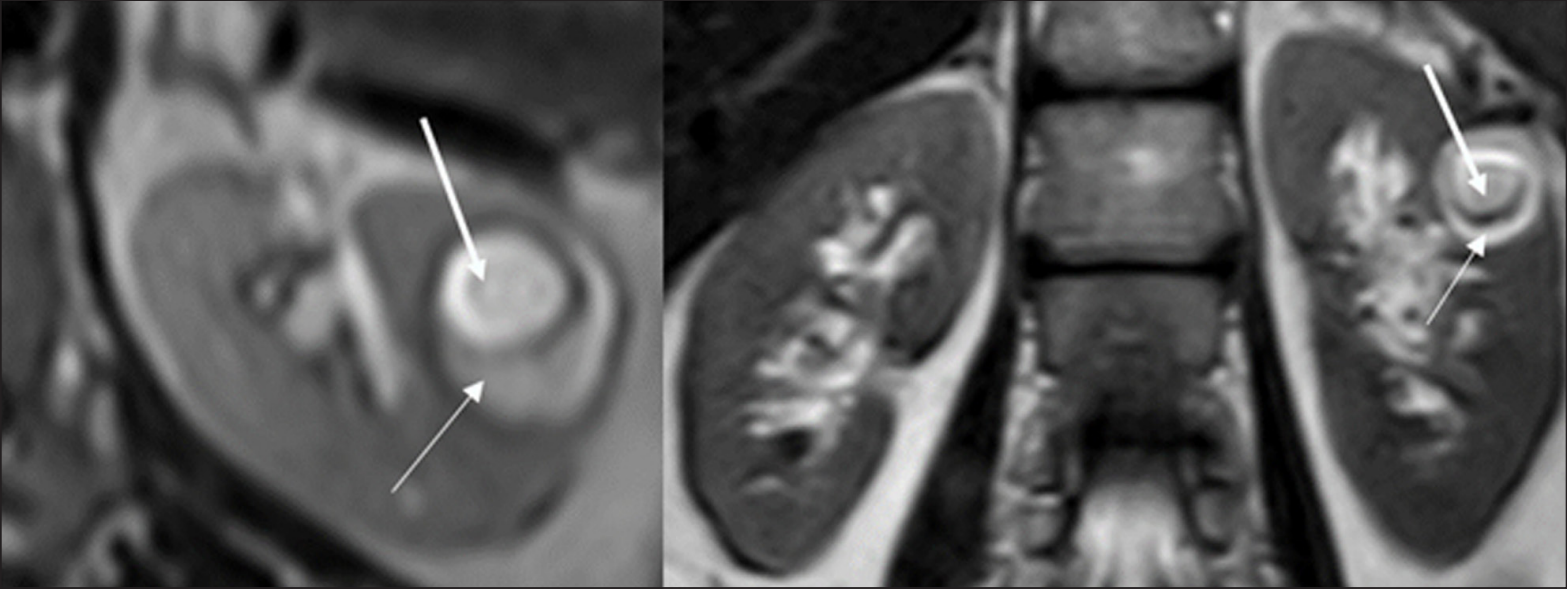


**Figure 3:**
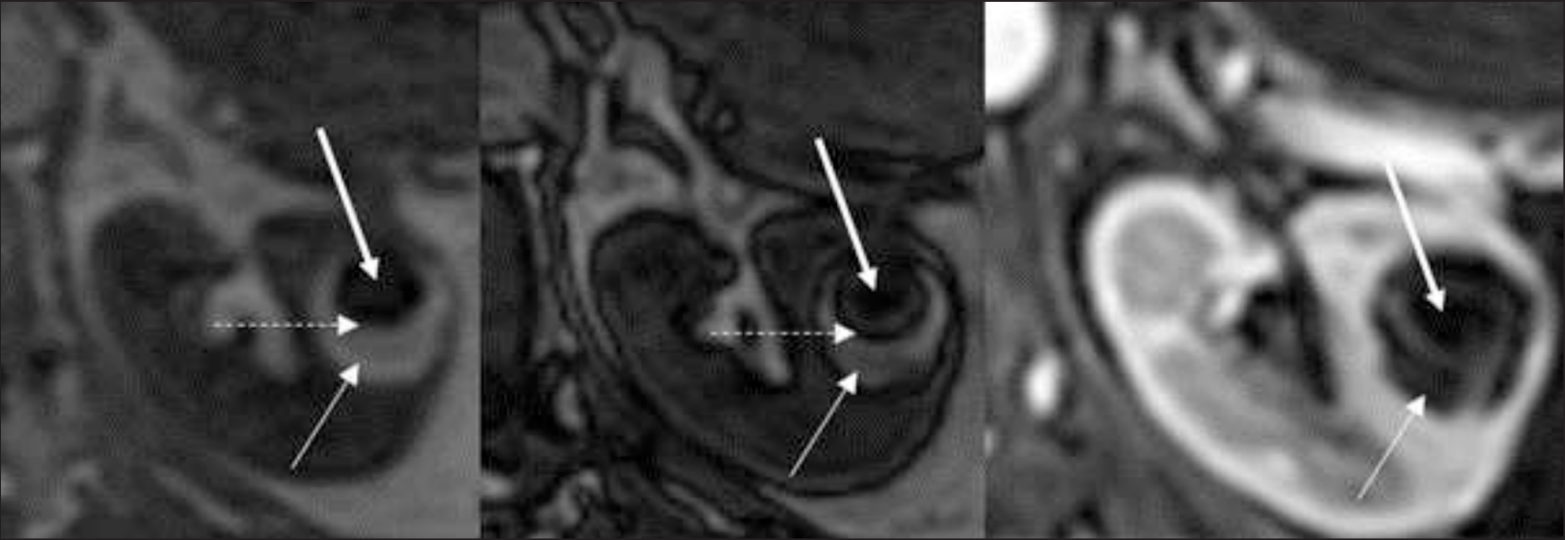


A 51-year-old man presented to his general physician with persistent mild abdominal pain. His prior history and family history were unremarkable. The patient did not use medication. Clinical examination and routine peripheral blood tests were normal. A screening abdominal ultrasound examination was requested to reassure the patient and showed no relevant findings regarding the abdominal pain. However, a hyperechoic 22 mm well-circumscribed donut-like lesion was visualized in the upper pole of the left kidney, with presence of a central cyst (Figure 1, arrows hyperechoic zone, dashed arrow cyst). Additional renal magnetic resonance imaging (MRI) confirmed the presence of a central cystic lesion in the left kidney upper pole with a surrounding spontaneous T1 hyperintense, T2 hyperintense soft tissue component that lost all signal when performing a fat saturated sequence (Figures 2 and 3, arrow fatty soft tissue, bold arrow cyst). At the interface of the cyst and the soft tissue component a rim of Indian ink artifact with discrete enhancement was seen (Figure 3, dashed arrow Indian ink artifact). No intralesional diffusion restriction or pathological enhancement was present. These findings are consistent with a fat-rich angiomylipoma with epithelial cyst (AMLEC).

## Comments

Angiomyolipomas (AMLs) are the most common benign renal tumours and are composed of vascular, smooth muscle and fatty elements. The presence of macroscopic fat within a lesion is the hallmark feature of AMLs on all modalities, but the amount of fat in these lesions may vary. Therefore, AMLs are radiologically divided into fat-rich, fat-poor, and fat-invisible subtypes. Due to these subgroups, imaging can differentiate between benign lesions (fat-rich) and lesions that require more careful evaluation (fat-poor and fat-invisible) [1].

In our case a benign fat-rich AML was diagnosed complicated with an epithelial cyst. Care should be taken not to confuse this AML with a primary cystic renal lesion, as the management and therapeutic options between an atypical renal cyst and AML differ significantly. Cysts are managed according to the Bosniak classification, while the AML approach depends on the symptomatology, the size of the lesion (cut-off 4 cm), and the diagnostic certainty on imaging. When possible, conservative treatment with active surveillance is preferred to maintain as many nephrons as possible. In other cases, AML can be treated with surgery, embolization, and ablation or in selected hereditary cases also with medication (mTOR inhibitors) [1].
